# Strukturelle Diskriminierung und Rassismus in der Krankenhausversorgung: die Rolle ökonomischer Rahmenbedingungen in der interkulturellen Öffnung

**DOI:** 10.1007/s00103-022-03615-x

**Published:** 2022-10-28

**Authors:** Steffen Schödwell, Mihaela Savin, Anke Lauke, Ingar Abels, Dana Abdel-Fatah, Simone Penka, Ulrike Kluge

**Affiliations:** 1 Zentrum für Interkulturelle Psychiatrie und Psychotherapie (ZIPP)/AG Transkulturelle Psychiatrie, Klinik für Psychiatrie und Psychotherapie, Charité Campus Mitte, Charité – Universitätsmedizin Berlin, Charitéplatz 1, 10117 Berlin, Deutschland; 2 TransVer – Ressourcen-Netzwerk zur interkulturellen Öffnung, Klinik für Psychiatrie und Psychotherapie, Charité Campus Mitte, Charité – Universitätsmedizin Berlin, Berlin, Deutschland; 3 Mentoring Competence Centers (MCC), Charité Campus Mitte, Charité – Universitätsmedizin Berlin, Berlin, Deutschland; 4 Berliner Institut für empirische Integrations- & Migrationsforschung (BIM), Kultur‑, Sozial- und Bildungswissenschaftliche Fakultät, Humboldt Universität zu Berlin, Berlin, Deutschland

**Keywords:** Rassismus, Strukturelle Diskriminierung, Kulturalisierung, Intersektionalität, Ökonomisierung, Racism, Structural discrimination, Culturalization, Intersectionality, Economization

## Abstract

**Hintergrund:**

Rassismus und strukturelle Diskriminierung in der deutschen Gesundheitsversorgung sind bislang wenig untersucht, obwohl die interkulturelle Öffnung seit vielen Jahren gefordert wird. Gleichzeitig schreiten die Prozesse der Ökonomisierung vor allem in der Krankenhausversorgung voran. Die vorliegende Studie untersucht aktuelle Herausforderungen der interkulturellen Öffnung unter Berücksichtigung der ökonomischen Rahmenbedingungen.

**Methoden:**

Es wurden 112 leitfadengestützte Interviews mit Mitarbeitenden der Berliner Krankenhausversorgung aus verschiedenen Berufsgruppen und Fachbereichen geführt. Gefragt wurde nach Herausforderungen, deren Bewältigung und Lösungsideen.

**Ergebnisse:**

Folgeerscheinungen der Ökonomisierung werden besonders in der Versorgung von Patient*innen mit Flucht‑/Migrationsgeschichte sichtbar. Ressourcenmangel kombiniert mit fehlender Kostenübernahme für Sprachmittlung führen zu Überforderung und zu einer Tendenz von Kulturalisierung, bei der die „Kultur“ der Patient*innen zur Erklärung ihres Handelns herangezogen wird, sowie von offenem Rassismus. Nährboden sind dabei multiple Verunsicherungen, die Mitarbeitende durch Mehrbedarfe dieser Patient*innen erleben. Kulturalisierung wird beschrieben als Bewältigungsversuch emotionaler Bedrängnis angesichts von Zeitmangel und Personalknappheit. In erster Linie wird daher der Wunsch nach mehr Zeit und Personal geäußert. Maßnahmen zum Abbau von Rassismus und struktureller Diskriminierung werden konkretisiert.

**Diskussion:**

Um Rassismus und Kulturalisierung entgegenzuwirken, sind Maßnahmen zentral, die an den ökonomischen Rahmenbedingungen sowie institutionellen Veränderungsprozessen ansetzen.

## Abstract (en)

**Background:**

Racism and structural discrimination in German healthcare have been little studied to date, although intercultural openness has been demanded for many years. At the same time, the processes of economization are progressing, especially in hospital care. This study examines current challenges of intercultural openness, including the influence of the economic framework.

**Methods:**

One hundred twelve guided interviews were conducted with hospital employees in Berlin from various professional groups and departments. They were asked about challenges, how they were coping, and ideas for solutions.

**Results:**

The consequences of economization are particularly visible in the care of patients with a history of flight or migration. A lack of resources combined with a lack of funding for language mediation leads to overburdening of healthcare workers and the tendency toward culturalization, in which the “culture” of the patients is used to explain their actions, and open racism. The breeding ground for this is the multiple insecurities experienced by staff due to the additional needs of these patients. Culturalization is described as an attempt to cope with their emotional distress in the face of lack of time and staff shortages. First and foremost, the wish for more time and additional staff was voiced. This being granted, further measures to reduce racism and structural discrimination were defined.

**Discussion:**

In order to counteract racism and culturalization, measures that focus on the economic framework conditions and institutional change processes are central.

## Einleitung

In unserer Studie wurde der Frage nachgegangen, wie es um die interkulturelle Öffnung der Berliner Krankenhäuser steht, welche fortbestehenden Barrieren sich identifizieren und welche Lösungsansätze sich ableiten lassen. Ziel der interkulturellen Öffnung ist es, eine gleichwertige und gleichberechtigte Versorgung von Menschen mit Migrationsgeschichte zu erreichen [1]. Hierfür werden mit dem Konzept umfassende Schritte der Personal‑, Organisations- und Qualitätsentwicklung eingefordert [2]. Trotz vielfacher Bestrebungen stoßen Menschen mit Flucht‑/Migrationsgeschichte weiterhin auf erhebliche Barrieren zur und innerhalb der Gesundheitsversorgung [2–6], die zu Fehl‑, Unter- und Überversorgung führen [2, 7, 8]. Diese sind neben Sprachbarrieren z. B. unterschiedliche Erklärungsmodelle hinsichtlich Gesundheit, Krankheit und Heilung, mangelhafte Informationen und Misstrauen gegenüber Versorgungsangeboten sowie Rassismus und strukturelle Diskriminierung [5]. Dabei werden Rassismus und strukturelle Diskriminierung in Deutschland über das Konzept der interkulturellen Öffnung allenfalls indirekt adressiert [9].

In den Sozialwissenschaften wird der Begriff „Rasse“ als ein soziales Konstrukt eingeordnet, das historisch der Rechtfertigung von Ausbeutung und Gewalt gegen Menschen zur Zeit des Kolonialismus diente, indem diese als „nicht weiß“ und damit als minderwertig rassifiziert wurden [10–12]. Die in der medizinischen Forschung bestehende biologische Nutzung als Ordnungskategorie ist mittlerweile falsifiziert [13–15]. Aktuelle sozialwissenschaftliche Auseinandersetzungen verstehen Rassismus hingegen als ein komplexes System, das auf unterschiedlichen Niveaus eine Hierarchisierung sowie Ausgrenzung (re)produziert [16–18]. Folgende Ebenen können unterschieden [19] und auf das Gesundheitssystem angewendet werden:

- *Makro-Ebene*: politische und ökonomische Rahmenbedingungen des Gesundheitssystems;
- *Meso-Ebene*: Versorgungsinstitutionen, ihre Versorgungs- und Verwaltungsroutinen;
- *Mikro-Ebene*: Denken, Fühlen und Handeln der einzelnen Mitarbeitenden.

Seit dem Zweiten Weltkrieg wurde beobachtet, dass „Kultur“ verstärkt zum signifikanten Differenzmarker geworden ist [20].

Im Rahmen des Models „racism without race“ hat Balibar [20] gezeigt, dass die Charakterisierung und Hierarchisierung sozialer Gruppen auf sozialen Grenzmarkierungen beruhen kann, die Kultur und Religion einschließen. Hieraus ergeben sich Verbindungspunkte zwischen dem Diskurs um Rassismus und dem zur interkulturellen Öffnung, in welchem die sogenannte Kulturalisierung als spezifische Form von Rassismus problematisiert worden ist. Dabei handelt es sich um ein Hervorheben von „Kultur“ als zentrale Begründung des Handelns von Personen, die gleichzeitig mittels des „Übersehens“ anderer Merkmale homogenisiert und stereotypisiert werden [21, 22]. Grundlage einer Kulturalisierung ist der Rückgriff auf einen statischen Kulturbegriff, indem der Veränderungscharakter von Kultur sowie der Umstand, dass Menschen sich hybrid mehreren Kulturen zugehörig fühlen können, ignoriert wird [23].

Rassismus und Kulturalisierung werden empirisch oft als unbewusste Voreingenommenheit (Implicit Bias) betrachtet und untersucht, ihre negativen Folgen für Behandlungszugänge, Diagnosen und Interventionen sind nachgewiesen [24–28]. Auf der Ebene von Aus‑, Fort- und Weiterbildungen sind kulturalisierende Haltungen, etwa indem sogenannte Kulturstandards/-wissen vermittelt werden, problematisiert worden [29, 30]. Interventionen, die darauf abzielen, Rassismus und strukturelle Diskriminierung im Gesundheitswesen zu reduzieren, setzen vornehmlich darauf, Mitarbeitende für ihren Implicit Bias zu sensibilisieren [31, 32]. Menschen und ihre Bedarfe sollten, anstelle eines Fokus auf kulturelle Differenz, vielmehr aus einer intersektionalen Perspektive betrachtet und Dimensionen gesellschaftlicher Ungleichheit wie Gender, Alter, Kultur/Ethnizität^1^, soziale Klassen und Bildung miteinander verschränkt berücksichtigt werden [33–36].

Parallel zur Forderung nach einer interkulturellen Öffnung sind in der deutschen Gesundheitsversorgung eine fortschreitende Ökonomisierung und Privatisierung der Krankenhausversorgung zu beobachten [37]. Als Zäsuren gelten das 1992 eingeführte Selbstkostendeckungsprinzip für Krankenhäuser sowie die Implementierung des DRG-Systems^2^ im Jahr 2003, infolgedessen ausschließlich Diagnosen und medizinisch-pflegerische Prozeduren als wichtige Kenngrößen den Behandlungsprozess sowie die Liegedauern steuern. Negative Folgen für die Arbeitsbedingungen der Mitarbeitenden und die Patient*innenversorgung sind entsprechend problematisiert worden [37].

## Methoden

Die Studie wurde im Rahmen des Projektes „TransVer neXus – Interkulturelle Öffnung der Berliner Krankenhäuser“ umgesetzt. Ziel des Projektes war, Herausforderungen in der Krankenhausversorgung von Menschen mit Flucht‑/Migrationsgeschichte zu identifizieren und anschließend durch konkrete Maßnahmen zu adressieren.

Von September 2020 bis Januar 2021 wurden zu diesem Zwecke 112 leitfadengestützte Interviews mit Mitarbeitenden aus Berliner Krankenhäusern durchgeführt. Zuvor wurden 520 Vertreter*innen aus möglichst allen Status- und Berufsgruppen zur Teilnahme eingeladen. Die Befragten, die an 7 berlinweiten Standorten und 5 somatischen sowie psychiatrisch-psychosomatischen Fachdisziplinen arbeiten, waren 77 Ärzt*innen, 19 Pflegefachkräfte, 7 Sozialarbeiter*innen, 7 Psycholog*innen sowie 2 Mitarbeitende aus dem kaufmännischen Bereich.

Die Konstruktion des Interviewleitfadens, das Führen sowie die Dokumentation und die Auswertung der Interviews erfolgten in einem multidisziplinären Team bestehend aus 4 Mitarbeiter*innen sowie 2 Leiterinnen in supervisorischer Funktion mit akademischer Ausbildung in Sozialwissenschaften, Anthropologie, Psychologie und Ethik.

Folgende Leitfragen strukturierten das Gespräch:

- „Welche Herausforderungen sehen Sie in der Versorgung von Patient*innen mit Flucht- und Migrationsgeschichte?“
- „Wie gehen Sie damit um? Was läuft bereits gut?“
- „Welche Verbesserungswünsche und -ideen haben Sie?“

Die 25- bis 60-minütigen Interviews führte jeweils ein*e Mitarbeiter*in per Telefon oder Video-Call durch. Kernaussagen, markante Zitate und Eindrücke zur Gesprächsatmosphäre wurden in Protokollen festgehalten. Vorläufige Beobachtungen und Interpretation wurden, wenn möglich, mit den Interviewten im Gespräch abgeglichen, bevor sie im Protokoll vermerkt wurden. Aufgrund der vollständigen Anonymisierung war eine schriftliche Einverständniserklärung nicht notwendig. Dokumentiert wurden lediglich Berufsgruppe, Statusebene (mit/ohne Leitungsfunktion) sowie eine Selbsteinordnung in 3 Fachbereichs-Cluster: „Psychiatrie und Psychosomatik“; „Somatik I“ (weniger kommunikationsintensiv) und „Somatik II“ (kommunikationsintensiv; Tab. 1).

**Table 1:** 

	Ärzt*innen	Psycholog*innen	Pflegekräfte	Sozialarbeiter*innen	Kaufmännischer Bereich	Gesamtzahl
*Anzahl*	*77*	*7*	*19*	*7*	*2*	*112*
Davon mit Leitungsfunktion	47	2	14	0	2	65
Psychiatrie und Psychosomatik	9	2	3	4	–	18
Somatik I	37	–	11	2	–	50
Somatik II	31	5	4	1	–	41
Keine Versorgung	–	–	1	–	2	3

Die Auswertung der Protokolle orientierte sich an der thematischen Analyse [38] und wurde durch die Datenanalysesoftware MAXQDA [39] unterstützt. Im ersten Schritt wurden bei der Hälfte der Protokolle die festgehaltenen O‑Töne, Aussagen oder Beobachtungen in Sinneinheiten unterteilt bzw. als separate Codings markiert und anschließend zur Systematisierung und Abstraktion mit einem vorläufigen Code versehen. Bei vergleichbarem Sinngehalt wurden einzelne Codings demselben Code zugeordnet. Diese wurden zunächst in der Gruppe diskutiert und nach inhaltlichen Überschneidungen sowie Sinnzusammenhängen zu einem Kategoriensystem mit Kernthemen und Codes strukturiert. Die restlichen Protokolle wurden im Anschluss ausgewertet und das vorläufige Kategoriensystem angepasst (Tab. 2). Dem Gütekriterium der intersubjektiven Nachvollziehbarkeit [40] folgend, wurden über den Analyseprozess hinweg Gedanken zum Inhalt und zur Struktur des Kategoriensystems festgehalten, im Team besprochen und konsentiert.

**Table 2:** 

Themen- bzw. Codenamen
*Zusammenspiel von Rahmenbedingungen, klinikinternen Verfahrensweisen und individueller Bewältigung*
Makro-Ebene: Ökonomisierung und fehlende Sprachmittlung
Meso-Ebene: klinikinterne Haltungen und Kompromisse
Mikro-Ebene: Frustration, Einzelkämpfer*innentum, Ablehnung und Rückzug
*Verunsicherungen bei Überforderung fördern Alltagsrassismen*
Kultureller Hintergrund
Religion
Geschlechterrollenerwartungen
Bildung
*Zuspitzungen führen zu Konflikten und Rassismusvorwürfen*
Eskalationen und aggressive Konflikte in Grenzsituationen
Abwertende(s) Verhalten und Gedanken von Mitarbeitenden
Rassismusvorwürfe ans Krankenhauspersonal
Diskriminierung durch Patient*innen
*Lösungsideen und -ansätze*
Makro-Ebene: mehr Ressourcen als Voraussetzung
Meso-Ebene: interkulturelle Öffnung als Leitstrategie
Mikro-Ebene: Fortbildung, Sensibilisierung und Empowerment

## Ergebnisse

Im Folgenden werden die Ergebnisse zu den vier Kernthemen und den zugehörigen Codes beschrieben. Beispielzitate und Protokollausschnitte bebildern die zusammenfassende Darstellung der Ergebnisse und lassen die Abstraktionsschritte zwischen Interviewmaterial und der Analyse sichtbar werden.

### Zusammenspiel von Rahmenbedingungen, klinikinternen Verfahrensweisen und individueller Bewältigung

Die Interviews verdeutlichen, wie die ökonomischen Rahmenbedingungen und die daraus resultierende Ressourcenknappheit mit klinikinternen Haltungen und Verfahrensweisen komplex zusammenhängen und die Ablehnung und Diskriminierung von Patient*innen mit Flucht-/Migrationsgeschichte fördern können. Um diese Verflechtung darzustellen, werden die Ergebnisse im Folgenden nach Zugehörigkeit zur Makro‑, Meso- und Mikro-Ebene präsentiert.

#### Makro-Ebene: Ökonomisierung und fehlende Sprachmittlung.

Die Befragten problematisieren den Personalnotstand sowie die zeitliche Taktung und die pauschalisierten Liegedauern des DRG-Systems. Durchgehend wird die fehlende Kostenübernahme von Sprachmittlung kritisiert. Im Fall von Geflüchteten werden zudem Unsicherheiten bzgl. Zuständigkeiten der Kostenübernahme von Gesundheitsleistungen sowie sich ggf. verändernder Regelungen des Asylsystems geäußert. Prekäre Lebensbedingungen von Asylbewerber*innen, wie eine Unterbringung in Gemeinschaftsunterkünften mit Mehrbettzimmern oder unsichere Aufenthaltsbedingungen, sehen die Befragten als ursächlich für deren mangelnden Zugang zur Gesundheitsversorgung und den oft geringeren Behandlungserfolg.

„Meine Rolle ist, die schwarze Null hinzubekommen. Wenn man die Dolmetscherkosten irgendwo auslagern wollen würde, würden die Krankenkassen sagen, dass das doch im DRG-System abgebildet ist. Das stimmt aber nicht“ (kaufmännischer Bereich mit Leitungsfunktion, Somatik I).

„Das stört mich an der Politik des ‚Wir schaffen das!‘: dass dieser Mehraufwand in den Kliniken in keiner Weise honoriert wird. Das muss ja nicht nur mit Geld passieren“ (Arzt*Ärztin mit Leitungsfunktion, Somatik II).

#### Meso-Ebene: Klinikinterne Haltungen und Kompromisse.

Es wird deutlich, dass sich Personal- und Zeitmangel, aber auch die sich aus dem DRG-System ergebenden Engpässe je nach Klinik bzw. Station erheblich unterscheiden. Aufgrund der fehlenden Regelung der Kostenübernahme von Sprachmittlung wird der Einsatz von Dolmetschenden zu einer Frage der Haltung der Klinikleitung. Während in einigen Versorgungsbereichen bzw. Kliniken eine Sprachmittlung aus den im DRG-System festgelegten Fallpauschalen finanziert und damit auf Einnahmen verzichtet wird, herrscht in anderen die Auffassung vor, dass Patient*innen für die Bewältigung der Sprachbarriere selbst verantwortlich sind. Eine andere verbreitete Lösung sind Listen von Mitarbeiter*innen, die zur Übersetzung für bestimmte Sprachen angefragt werden können. Die Versorgung Geflüchteter stößt aufgrund der teilweise unsicheren Kostenübernahme bzw. des nicht ausreichend refinanzierten Mehraufwandes mitunter in der Leitungsebene auf Ablehnung.

„Der wirtschaftliche Druck ist enorm. Es wird von Jahr zu Jahr schlimmer. Der Frust wird auch bei allen Statusgruppen immer größer“ (Arzt*Ärztin, Somatik I).

„Momentan ist die Realität, dass Mitarbeitende zum Übersetzen herangezogen werden, auch Reinigungskräfte. Das geht nicht! Diese Lücken müssen geschlossen werden!“ (Arzt*Ärztin mit Leitungsfunktion, Somatik II).

#### Mikro-Ebene: Frustration, Einzelkämpfer*innentum, Ablehnung und Rückzug.

Die Befragten bringen allgemeine Überforderung, Ohnmacht und Frustration zum Ausdruck. Die Qualität dieses Erlebens unterscheidet sich abhängig von der Klinik, der Station und auch der Berufsgruppe. Zentrales Narrativ ist, dass Patient*innen mit Mehrbedarf „das Fass schnell zum Überlaufen bringen“ können. Deren Versorgung hängt daher häufig vom Engagement einzelner Mitarbeitender ab. Befragte aus der Pflege und Befragte ohne Leitungsfunktion waren dabei am stärksten belastet. Vor allem Mitarbeitende mit eigener Migrationsgeschichte können in Überforderung und Rollenkonflikte geraten, wenn sie als Sprachmittler*innen eine Zusatzfunktion übernehmen (müssen) oder von Kolleg*innen und Patient*innen als Expert*innen für „eine bestimmte Kultur“ oder Rassismus im Allgemeinen angefragt werden. Kritisch betrachtet wurde die Verhinderung struktureller Änderungen auf gesetzlicher und Klinik-Ebene sowie die daraus entstehende Individualisierung von Belastung.

Einige Befragte äußern Ablehnung gegenüber Patient*innen, deren Deutschniveau im Verhältnis zur Aufenthaltsdauer in Deutschland nicht hinreichend sei. Dieselben Mitarbeitenden betonen gleichzeitig negative Auswirkungen der Sprachbarriere auf die Behandlung sowie den Wunsch der Integration von Sprachmittlung als finanzierte, gut verfügbare Ressource in die Gesundheitsversorgung. Dies zeigt ihre Ambivalenz hinsichtlich der Rolle des Gesundheitssystems bzgl. Integrations- und Bildungsaufgaben, z. B. indem sie nachdenken, ob der Einsatz von Sprachmittlung in der Gesundheitsversorgung dazu beiträgt, dass Patient*innen nicht hinreichend Deutsch lernen. Die Befragten sehen sich zudem mit ethischen Dilemmata konfrontiert, wenn Behandlungen aufgrund mangelnder Kommunikationsmöglichkeiten ggf. eingeschränkt werden müssen. Andererseits wird es als frustrierend erlebt, wenn erbrachte Gesundheitsleistungen, wie z. B. im Falle von Nichtversicherten, nicht refinanziert werden.

„Ehrlich gesagt gibt’s die [versorgungserleichternden Maßnahmen] nicht. Man ist auf sich selbst gestellt. Man muss selber klarkommen“ (Pflege, Somatik I).

„Was aggressiv macht, ist die Überforderung, die jede Berufsgruppe unterschiedlich [gut] kompensieren kann“ (Arzt*Ärztin, Somatik I).

„Die meisten Leute, die ich treffe, sind sehr nette Leute, die wollen mit und am Menschen arbeiten, aber sie haben nicht die Zeit, um das zu tun. Wenn ich ständig übermüdet bin und es piept und so weiter, dann werde ich auch aggressiv. Ich bin vorsichtig, das dann rassistisch zu nennen. Es ist in dem Augenblick die einzige gangbare Strategie, das Pensum zu schaffen: Man wird ruppig“ (Arzt*Ärztin, Somatik II).

### Verunsicherung bei Überforderung fördert Alltagsrassismen

Mitarbeitende sprechen von deutlichen Verunsicherungen in der Behandlung von Menschen mit Flucht‑/Migrationsgeschichte. Einige verorten diese vornehmlich auf kultureller Ebene, andere nehmen eine intersektionale Perspektive ein und berücksichtigen Religion, Bildung oder Geschlechterrollenerwartungen mit. Die Verunsicherungen verstärken sich im Kontext von Personal- und Zeitmangel und/oder fehlender Sprachmittlung zu Überforderung und Frustration.

Befragte ohne Migrationsgeschichte fühlen sich durch Kolleg*innen mit eigener Migrationserfahrung, die Einblicke in die jeweilige „Kultur“ der Patient*innen geben können, entlastet. Befragte mit Migrationserfahrung berichten hingegen von Überforderung und Rollenkonflikten. Teils nehmen Mitarbeitende selbstreflexiv die Tendenz wahr, Komplexität und Spannung durch Stereotypisierungen, Kulturalisierung und Ablehnung zu reduzieren. Einige unterscheiden „tatsächlichen Rassismus“ von überforderungsbedingter Ablehnung.

#### Kultureller Hintergrund.

Eine Vielfalt an Herkunftsregionen, oft gleichgesetzt mit großer kultureller Diversität, wird mit einem Mehr an benötigten Kompetenzen assoziiert. Ebenso werden bei Patient*innen mit Migrationsgeschichte Unterschiede in Affekt- und Symptomerleben bzw. dessen Ausdruck (z. B. von Schmerzen) wahrgenommen. Teils als kulturbedingt problematisiert werden auch eine verstärkte Präsenz und aktive Teilnahme der Familie an Diagnose und Therapie. Mitarbeitende erleben Mehraufwand und Konflikte bzgl. des Durchsetzens von Besuchsregeln sowie in der Kommunikation über Behandlungswünsche und -bedürfnisse.

„Familienbesuch ist auch ein großes Thema; bei „deutschen“ Patient*innen kommen ein bis zwei Leute, bei [Nationalität] Patient*innen kommen 10–20 und halten den gesamten Ablauf der Station auf. Dann ist das Team immer regelmäßig sehr frustriert, weil es schwer ist zu arbeiten und weil es dem Team zu laut ist. Dann fallen Sätze wie ‚So geht das hier aber nicht, wir sind doch hier in Deutschland.‘“ (Arzt*Ärztin, Somatik II).

#### Religion.

Glaube und Spiritualität von Patient*innen werden zum Teil als konfliktfördernd in Bezug auf klinische Abläufe wahrgenommen. Vor allem muslimische Patient*innen werden häufig aufgrund von Ernährung, regelmäßiger Gebetspraxis und Bekleidung zu „Sonderfällen“.

#### Geschlechterrollenerwartungen.

Verunsicherung und Spannungen werden auch im Umgang mit Familienstrukturen berichtet, die von Befragten als „patriarchal“ beschrieben werden. Einige sehen diese als Teil der „Kultur“ von Patient*innen an, andere als Differenzen in Geschlechterrollenerwartungen. Besonders die Nichtakzeptanz von Behandlerinnen durch männliche Patienten wird als konflikthaft erlebt. Während der Wunsch von Patient*innen nach geschlechtlicher Passung der behandelnden Person bei einigen Mitarbeitenden Unverständnis und Ärger auslöst, berichten andere, dass sie, wenn es personell möglich ist, auf diesen Wunsch selbstverständlich eingehen.

#### Bildung.

Einige Mitarbeitende betrachten ein wahrgenommenes niedrigeres Bildungsniveau sowie eine unzureichende Gesundheitskompetenz in Form von Unwissen bzgl. Körper und Gesundheitssystem als ursächlich für Schwierigkeiten in der Versorgung von Menschen mit Migrationsgeschichte. Als besonders herausfordernd gilt die Versorgung von Analphabet*innen, weil diese aufgrund der Notwendigkeit einer mündlichen Aufklärung gepaart mit Sprachmittlung deutlich mehr Zeit erfordert und zudem oftmals auf eine einfache Sprache geachtet werden muss. Häufig müssen Mitarbeitende Eigeninitiative aufbringen oder mit unzureichender Aufklärung Behandlungen durchführen, was rechtliche Verunsicherung, emotionale Anspannung und Ablehnung dieser Patient*innen begünstigt.

Bedingt der Fokus auf vermeintliche kulturelle Differenz oft den Wunsch nach mehr „Kulturwissen“, erhoffen sich Mitarbeitende, die Bildungsunterschiede wahrnehmen, z. B. Hilfe durch (übersetzte) Aufklärungsmaterialien. Andere beziehen aufgrund gegebener Geschlechterrollenerwartungen gezielt Familienmitglieder ein. Deutlich wird, dass eine solch differenzierte Betrachtung Zeit und zusätzliche Ressourcen braucht.

„… im Großen versuchen allerdings alle eine gute Lösung zu finden. Der ‚Goodwill‘ kippt unter Stress schnell zurück ins Vorurteil. Wenn da mehr Zeit wäre, würde das gehen. Das Problem ist, wenn es zu rigide wird. ‚So wird das aber hier nicht gemacht.‘ ‚Wir sprechen hier aber Deutsch‘, dann entstehen häufig Konflikte …“ (Protokollausschnitt, Psychologe*in, Somatik II).

### Zuspitzungen führen zu offenen Konflikten und Rassismusvorwürfen^3^

Berichtet wird bei chronischer Überforderung und/oder in zugespitzten Behandlungssituationen von teils verbalen oder körperlichen Konflikten mit Patient*innen, Angehörigen sowie in Teams, in denen sich Rassismus(vorwürfe) und Diskriminierung zeigen. Eskalationspunkte sind häufig Stationsregeln und Behandlungsmisserfolge bzw. Kommunikation über Therapien am Lebensende, also sogenannte Grenzsituationen. Dabei wiegt fehlende Sprachmittlung besonders schwer. Eine gelingende sprachliche Kommunikation sowie multiprofessionelle Zusammenarbeit mit gezieltem Einsatz von (ober-)ärztlicher Autorität, z. B. in der Kommunikation hinsichtlich Absprachen zum Stations- und Behandlungsablauf, können hingegen gegenseitiges Verständnis zwischen Mitarbeitenden und Patient*innen ermöglichen.

Weitere Manifestationen von Rassismus und Diskriminierung werden von Befragten sowohl bei sich selbst als auch im Verhalten anderer beobachtet:

- Gedanken, dass Patient*innen sich anpassen oder „nach Hause gehen“ sollten;
- Unwille, Patient*innen mit Flucht‑/Migrationsgeschichte und/oder ohne Krankenversicherungsschutz (mit kostenintensiven Therapien) zu behandeln;
- abwertende, ablehnende und feindselige Äußerungen (gegen-)über Patient*innen und Mitarbeitenden mit Migrationsgeschichte;
- Forderung vergleichsweise strengerer Handhabungen von Regelverstößen.

Im Klinikalltag scheint es kaum Raum zu geben, sich mit solchen schwierigen Themen zu beschäftigen. Einige Mitarbeitende äußern, dass sie bestimmte Erfahrungen lange mit sich herumtragen, z. B. wenn Patient*innen durch sie selbst oder durch andere Mitarbeitende diskriminiert wurden oder ihnen gegenüber Rassismusvorwürfe geäußert wurden.

„Man ertappt sich ja selbst! Als mir der [Nationalität] vor die Füße spuckte, hab ich auch gedacht: Dann geh doch zurück nach [Nation]! Da muss man sehr aufpassen!“ (Arzt*Ärztin mit Leitungsfunktion, Somatik II).

„Wenn keine gelenkte Nachbesprechung stattfindet, verstärken sich die Schubladen“ (Arzt*Ärztin mit Leitungsfunktion, Somatik I).

### Lösungswünsche und -ansätze

Die Befragten äußerten verschiedene Lösungsideen und -ansätze, um die strukturelle Diskriminierung in der Versorgung von Menschen mit Mehrbedarfen, darunter Menschen mit Flucht‑/Migrationsgeschichte, zu adressieren.

#### Makro-Ebene: Mehr Ressourcen als Voraussetzung.

An erster Stelle weisen die Befragten darauf hin, dass Personalmangel und Zeitknappheit sowie die fehlende Kostenübernahme von Sprachmittlung zuerst zu adressieren sind, anstatt auf spezifische Angebote zum Abbau von Rassismus und Diskriminierung zu setzen. Erst damit seien Voraussetzungen für eine interkulturelle Öffnung geschaffen. Sie wünschen sich eine bessere Steuerung und Flexibilisierung der verschiedenen Versorgungsstrukturen, z. B. eine bessere Verzahnung von ambulanter und stationärer Versorgung, sowie Zugänglichkeit von Informationen zu den einzelnen Angeboten für Patient*innen, z. B. dem Behandlungsangebot und -ablauf einer spezifischen Station. Ebenso formulierten sie den Wunsch nach einer besseren Vernetzung mit Kostenträgern und Institutionen der Geflüchtetenversorgung.

„Wenn ich mich entscheiden müsste Dolmetscher oder Personal, würde ich mich immer fürs Personal entscheiden“ (Arzt*Ärztin mit Leitungsfunktion, Somatik II).

#### Meso-Ebene: Interkulturelle Öffnung als Leitstrategie.

Auf klinikinterner Ebene fordern die Befragten von der Klinikleitung, die interkulturelle Öffnung stärker zu fördern und mittels Engagements und Schaffung struktureller Voraussetzungen eine bedarfsgerechte Versorgung von Menschen mit Flucht‑/Migrationsgeschichte zu gewährleisten. Hierzu zählen der Wunsch nach divers zusammengesetzten Teams, aber auch klare Finanzierungs- und Organisationsstrukturen zum systematischen Einsatz von schnell zugänglicher Sprachmittlung. Einige Befragte betonen die Notwendigkeit einer stärkeren Vernetzung mit psychosozialen Versorgungsstrukturen und Migrant*innenselbstorganisationen.

#### Mikro-Ebene: Fortbildung, Sensibilisierung und Empowerment.

Auf Mitarbeitenden-Ebene äußern die Befragten Bedarf nach Fort- und Weiterbildung, wobei die Schaffung zeitlicher Ressourcen für diese betont werden. Uneinigkeit besteht darüber, ob diese Angebote freiwillig oder verpflichtend sein sollten. Eindeutig ist der Wunsch nach Stärkung der Kommunikation und Zusammenarbeit im Team und der Etablierung regelmäßiger Formate, wie beispielsweise von Supervisionen zum Thema „Versorgung von Patient*innen mit Sprachbarriere“. Einige fragen nach Weiterbildungen, um spezifisches (Kultur‑)Wissen zu erlangen, andere wünschen eine allgemeine Sensibilisierung oder eine Vermittlung von allgemeinen kommunikativen Kompetenzen, wie z. B. in Deeskalationstrainings. Für Patient*innen fordern die Befragten Möglichkeiten der Information und Beratung über das Gesundheitssystem oder ihre Rechte.

Zusammenfassend formulieren die Mitarbeitenden auf klare Weise, dass es umfassender Maßnahmen bedarf, um Rassismus und strukturelle Diskriminierung in der Krankenhausversorgung abzubauen.

## Diskussion

Das Novum unserer Studie liegt darin, hinlänglich bekannte Folgeerscheinungen der Ökonomisierung [37], fortbestehende Sprachbarrieren [2] sowie die Tendenz zu Kulturalisierung bzw. unbewusster Voreingenommenheit (Implicit Bias) [21–26] in einen Zusammenhang zu bringen. Unsere Ergebnisse zeigen, dass sich die voranschreitende Ökonomisierung und der damit einhergehende Personal- und Zeitmangel besonders in der Versorgung von Menschen mit Mehrbedarfen zeigen. Menschen mit Migrations- und besonders Fluchtgeschichte werden zu „herausfordernden“ Patient*innen, die auf institutioneller und Leitungsebene auf eine ablehnende Haltung treffen können. Mitarbeitende kommen bei der Konfrontation mit institutionell zeitlich nicht vorgesehenen Versorgungsbedarfen in Bedrängnis, sich überdurchschnittlich oder gar nicht zu engagieren. Zentrale Beobachtung unserer Studie ist, dass die ökonomischen Rahmenbedingungen der Krankenhausversorgung, insbesondere das DRG-System, und die fehlende Kostenübernahme von Sprachmittlung somit bestehende Strukturen von Diskriminierung und Rassismus verstärken (können).

Ein kürzlich durchgeführtes Review über Rassismus im Gesundheitswesen hat gezeigt, dass die Forschung über Rassismus im Gesundheitswesen eher „beschreibend, ahistorisch und atheoretisch“ ist und sich kaum mit den Entstehungsprozessen von Rassismus auseinandersetzt. Sie befasst sich mehr mit der Untersuchung von rassischen Kategorien und behandelt diese häufig als „reale Kategorien“ [41]. In dieser Studie haben wir versucht, über die Beschreibung der bloßen Existenz von Rassismus hinauszugehen, indem wir die Frage beleuchteten, welche ökonomischen Strukturen die Existenz von Rassismus im Gesundheitssektor aufrechterhalten, eine Frage, die unserem Wissen nach im Bereich des Gesundheitswesens weitgehend vernachlässigt wird. Zur Untersuchung dieser Frage haben wir einen Analyserahmen verwendet, der zwischen der Makro‑, Meso- und Mikro-Ebene unterscheidet.

Unsere Studie legt die These nahe, dass die Gewinnorientierung im (DRG-)Gesundheitssystem eine gleichberechtigte Versorgung von Menschen mit Flucht‑/Migrationsgeschichte erheblich erschwert, weil die Versorgung von Mehrbedarfen nicht honoriert wird. Zudem schafft sie Rahmenbedingungen, in denen Klinikmitarbeitende aus emotionaler Bedrängnis auf Rassismen, Diskriminierung und Kulturalisierung zurückgreifen [42]. Wenig verwunderlich zeigt sich dies am deutlichsten in Situationen, in denen eine besonders große Verunsicherung und ein Mehrbedarf, z. B. an Zeit und Kommunikation, einer Verknappung von Ressourcen gegenüberstehen. Dies steht im Einklang mit Erkenntnissen, dass auf kognitiver Ebene (akuter) Stress zu stark vereinfachtem oder rigidem Denken sowie zu einem gesteigerten Rückgriff auf Automatismen, in diesem Falle Stereotypisierungen und Rassismen, führt [43, 44]. Die kognitive Anstrengung, die für eine Bewertung individueller Merkmale einer Person notwendig ist, ist größer als die Anstrengung bei der Anwendung von Gruppenmerkmalen [24].

Der Abbau von Rassismus und Diskriminierung im Gesundheitswesen sollte demnach von einer Veränderung der ökonomischen Rahmenbedingungen begleitet sein, in denen der Zeitaufwand in der Versorgung von Patient*innen mit Mehrbedarfen, darunter Menschen mit Flucht-/Migrationsgeschichte, realistisch abgebildet wird. Voraussetzungen für die Etablierung von rassismus- und diskriminierungssensibleren Strukturen und Verfahrensweisen auf Ebene der Kliniken, Fachabteilungen und Stationen sind demnach vor allem Veränderungen auf der Makro-Ebene. Lösungswünsche der Befragten benennen diesen Umstand in unmissverständlicher Weise.

Auf Mikro-Ebene wurde deutlich, dass Mitarbeitende die Bedarfe ihrer Patient*innen besser erfassen, wenn sie verschiedene Differenzierungsdimensionen (wie Bildung, Alter, Geschlecht etc.) und ihr Zusammenspiel in den Blick nehmen und hierdurch Kulturalisierung vorbeugen [36]. Strategien sollten nicht nur einseitig auf Fort- bzw. Weiterbildung der Mitarbeitenden setzen, sondern in strukturverändernde bzw. -bildende Maßnahmen auf Makro- und Meso-Ebene eingebettet sein. Damit Beschäftigte einen intersektionalen Blick einnehmen und verschiedene Differenzierungsdimensionen verschränkt betrachten, bedarf es, so verdeutlichen es die Ergebnisse unserer Studie, der entsprechenden Ressourcen.

### Limitationen

Trotz der großen Stichprobe erlaubt das qualitative Design der Studie keine Aussagen zur Häufigkeit der genannten Phänomene. Solche Quantifizierungen werden zudem durch die starke Tendenz, in Interviews sozial erwünscht zu antworten, begrenzt. Eine weitere Herausforderung ergibt sich aus dem Verzicht einer Tonbandaufzeichnung der Gespräche. Der Gefahr einer unsachgemäßen Vermischung von Datenerhebung und -interpretation in der Protokollierung wurde begegnet, indem vorläufige Hypothesen mit den Befragten abgeglichen wurden, bevor sie niedergeschrieben wurden.

## Fazit

Um Rassismus und strukturelle Diskriminierung abzubauen, muss nicht allein auf der Mikro-Ebene, sondern vor allem auch auf der Makro- und Meso-Ebene angesetzt werden, beispielsweise mit:

- einem besseren Personalschlüssel, der den Mehraufwand für Patient*innen mit Sprachbarrieren zeitlich auffängt,
- einer Finanzierung und dem systematischen Einsatz von leicht zugänglicher Sprach- und Kulturmittlung,
- einem Engagement der Leitungen, klinikinterne strukturelle Voraussetzungen zu schaffen,
- einer Etablierung von regelmäßigen Formaten zur (Konflikt‑)Verarbeitung und/oder Reflexion von Rassismus‑/Diskriminierungserfahrungen im klinischen Alltag innerhalb der Teams,
- einer Verankerung von Aus‑, Fort- und Weiterbildungsangeboten zu transkulturellen Kompetenzen mit Fokus auf Steigerung der Unsicherheitstoleranz sowie Sensibilisierung für sozioökonomische Aspekte von Flucht und Migration sowie Diskriminierung und Rassismus;
- dem Schaffen einer klinikinternen Beschwerdestelle für Rassismus und Diskriminierung.

## Footnotes

1 Trotz der benannten Kritik ist die Verwendung von Ethnizität/kultureller Hintergrund im deutschen Diskurs weiterhin üblich.

2 DRG (Diagnosis Related Groups): Das DRG-System ist ein pauschalisierendes Abrechnungssystem nach diagnosebezogenen Fallgruppen für stationäre Krankenhausbehandlungen.

3 Aufgrund einer vergleichsweise geringeren Datendichte wird dieses Kernthema abweichend von den anderen und Tab. 2 zusammenfassend und nicht unterteilt in Codes dargestellt.
