# Erratum zu: Strukturelle Diskriminierung und Rassismus in der Krankenhausversorgung: die Rolle ökonomischer Rahmenbedingungen in der interkulturellen Öffnung

**DOI:** 10.1007/s00103-022-03624-w

**Published:** 2022-12-06

**Authors:** Steffen Schödwell, Mihaela Savin, Anke Lauke, Ingar Abels, Dana Abdel-Fatah, Simone Penka, Ulrike Kluge

**Affiliations:** 1 Zentrum für Interkulturelle Psychiatrie und Psychotherapie (ZIPP)/AG Transkulturelle Psychiatrie, Klinik für Psychiatrie und Psychotherapie, Charité Campus Mitte, Charité – Universitätsmedizin Berlin, Charitéplatz 1, 10117 Berlin, Deutschland; 2 TransVer – Ressourcen-Netzwerk zur interkulturellen Öffnung, Klinik für Psychiatrie und Psychotherapie, Charité Campus Mitte, Charité – Universitätsmedizin Berlin, Berlin, Deutschland; 3 Mentoring Competence Centers (MCC), Charité Campus Mitte, Charité – Universitätsmedizin Berlin, Berlin, Deutschland; 4 Berliner Institut für empirische Integrations- & Migrationsforschung (BIM), Kultur‑, Sozial- und Bildungswissenschaftliche Fakultät, Humboldt Universität zu Berlin, Berlin, Deutschland

**Erratum zu:**

**Bundesgesundheitsbl 2022**

10.1007/s00103-022-03615-x

In der ursprünglichen Originalversion des Artikels wurde ein Satz in der Diskussion in Bezug auf ein kürzlich durchgeführtes Review über Rassismus im Gesundheitswesen nicht korrekt wiedergegeben.

Dort stand:

Ein kürzlich durchgeführtes Review über Rassismus im Gesundheitswesen hat gezeigt, dass die Forschung über Rassismus im Gesundheitswesen eher „beschreibend, historisch und theoretisch“ ist und sich kaum mit den Entstehungsprozessen von Rassismus auseinandersetzt.

Dies wurde korrigiert in:

Ein kürzlich durchgeführtes Review über Rassismus im Gesundheitswesen hat gezeigt, dass die Forschung über Rassismus im Gesundheitswesen eher „beschreibend, ahistorisch und atheoretisch“ ist und sich kaum mit den Entstehungsprozessen von Rassismus auseinandersetzt.

Im Zuge der Übersetzung des Zitats war der Fehler entstanden, welcher den Sinn der Aussage ins Gegenteil verkehrt hatte.

Der Originalbeitrag wurde korrigiert.

